# Corrigendum: Earliest signs of life on land preserved in ca. 3.5 Ga hot spring deposits

**DOI:** 10.1038/ncomms16149

**Published:** 2017-08-16

**Authors:** Tara Djokic, Martin J. Van Kranendonk, Kathleen A. Campbell, Malcolm R. Walter, Colin R. Ward

Nature Communications 8: Article number: 15263; DOI: 10.1038/ncomms15263 (2017); Published: 05 09 2017; Updated: 08 16 2017.

The original manuscript presented evidence that the deposits studied were fluvial rather than marine to support the conclusion that evidence of life on land had been discovered in the 3.4 Ga Dresser Formation, Pilbara Craton, Australia.

The authors acknowledge that the following reference, which was omitted, should have been cited to provide more detailed sedimentological and stratigraphic evidence that the deposits studied were indeed fluvial rather than marine.

Djokic, T. *Assessing the Link Between Earth’s Earliest Convincing Evidence of Life and Hydrothermal Fluids: The c. 3.5 Ga Dresser Formation of the North Pole Dome, Pilbara Craton, Western Australia* (MPhil thesis, The University of New South Wales, 2015).

