# Supplementary material for: Earliest signs of life on land preserved in ca. 3.5 Ga hot spring deposits
Source: Nat Commun. 2017 May 9;8:15263. doi: 10.1038/ncomms15263 (PMC5436104; doi:10.1038/ncomms15263)
Supplement: Supplementary Information — Supplementary Figures [file ncomms15263-s1.pdf]

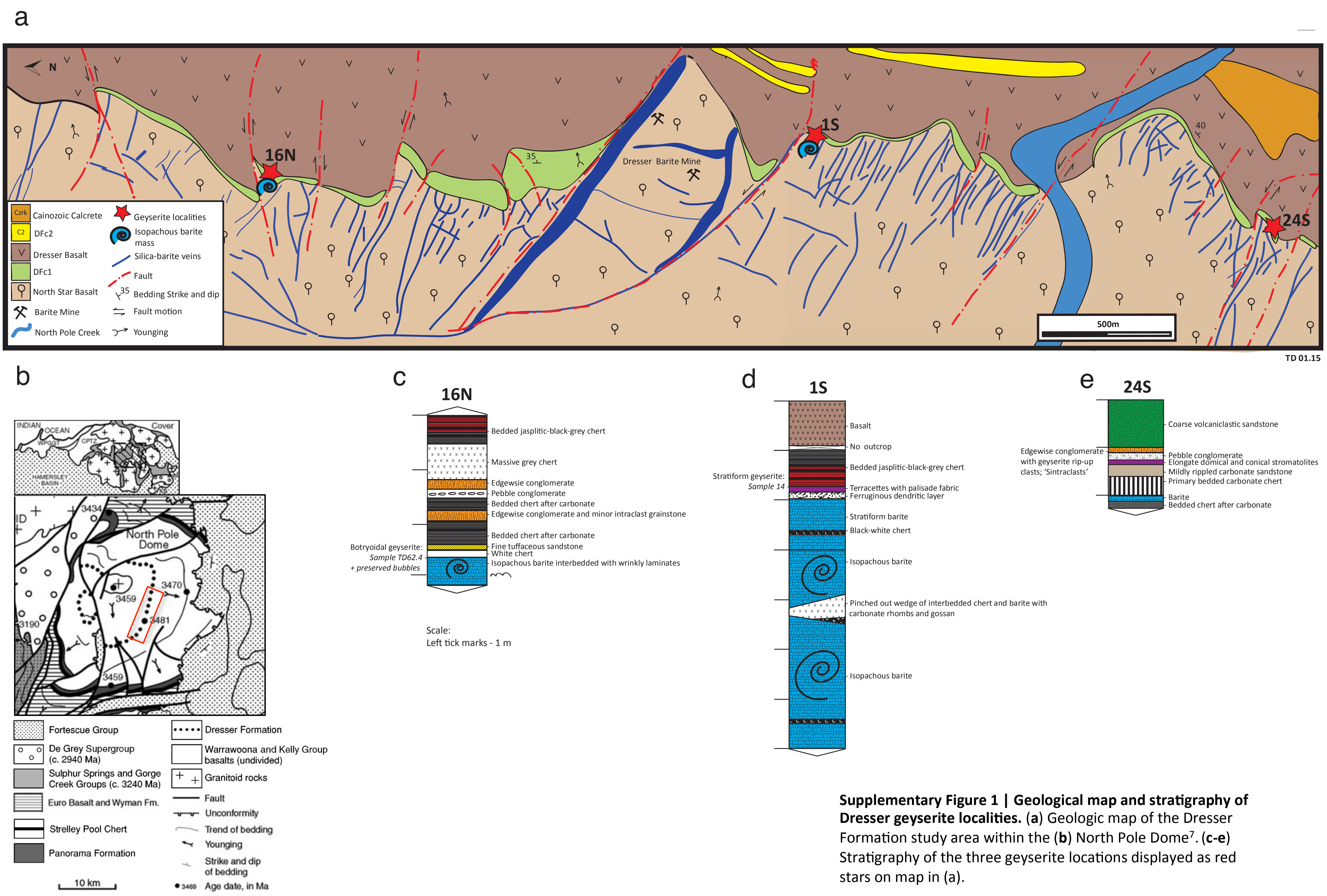

Supplementary Figure 1 | Geological map and stratigraphy of Dresser geyserite localities. (**a**) Geologic map of the Dresser Formation study area within the (**b**) North Pole Dome[7]. (**c-e**) Stratigraphy of the three geyserite locations displayed as red stars on map in (a).

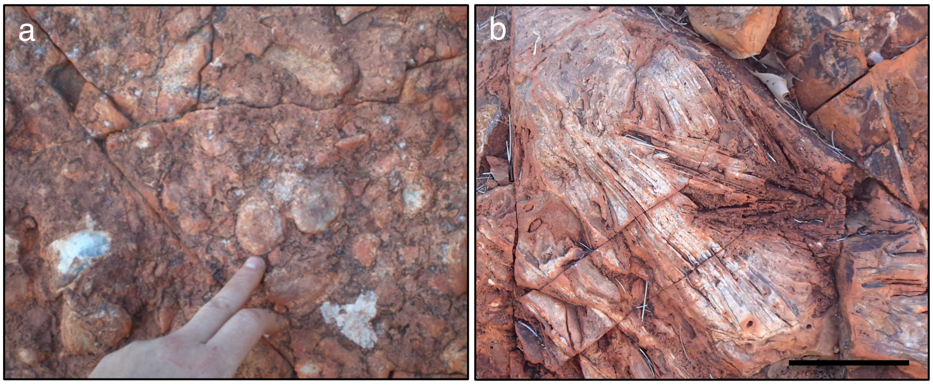

Supplementary Figure 2 | Locality 24S containing Dresser geyserite sintraclasts in an inferred fluvial setting. (**a**) Pebble to cobble conglomerate. (**b**) Edgewise conglomerate with sinter clasts stacked in packed arrays. (Scale bar: 10 cm).

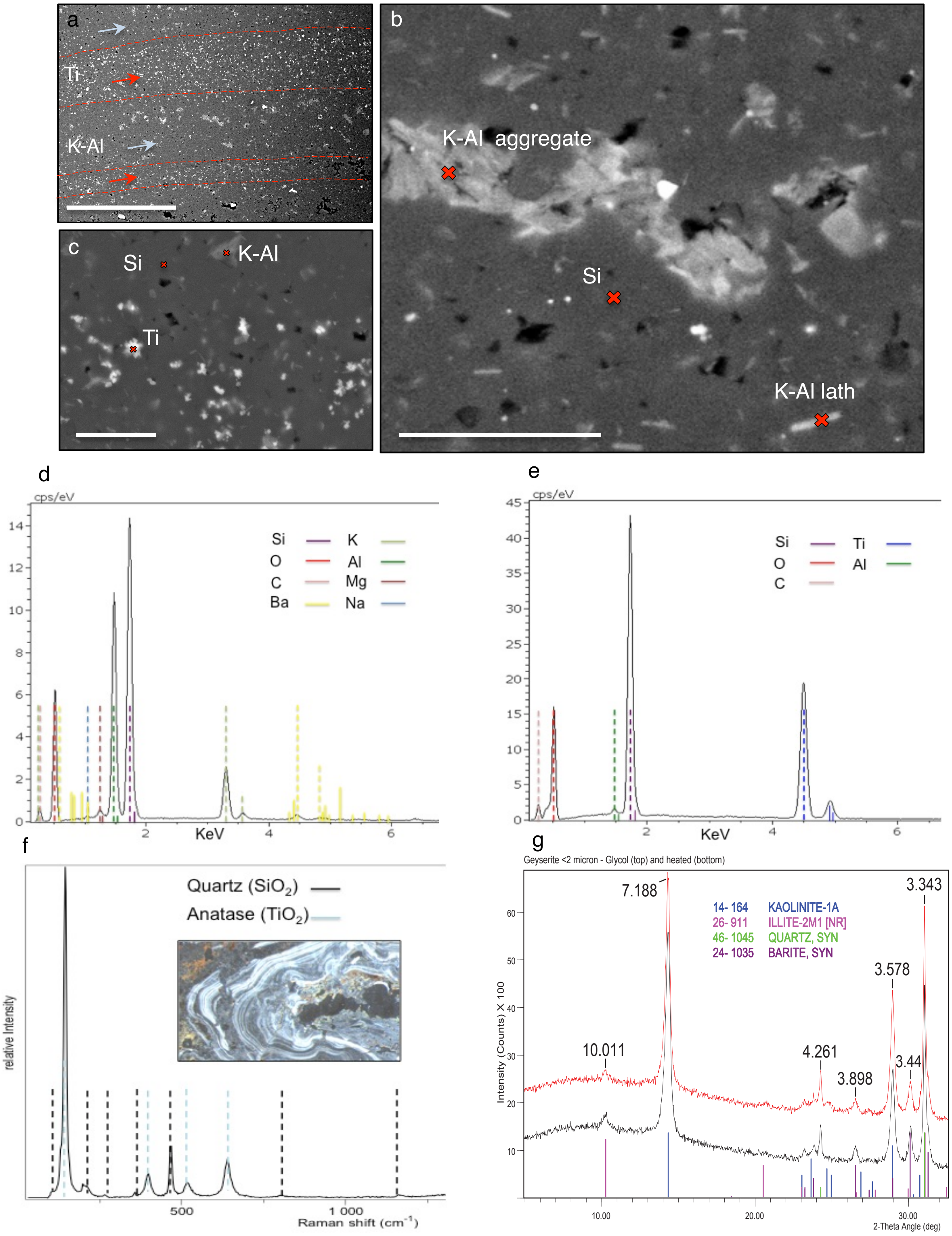

Supplementary Figure 3 | Mineral data of Dresser botryoidal geyserite (Location 16N). Scale bar measurements indicated. (**a**) BSE image of K-Al rich flakes in light layers and Ti-rich crystals concentrated in dark layers, set in a Si (microquartz) matrix. (100 µm). (**b**) BSE image of K-Al (kaolinite+illite) laths and flakes. (10 µm). (**c**) Close-up of TiO_2 (anatase) grains. (10 µm). (**d**) SEM-EDS spectra for Al-K flakes in (b). (**e**) SEM-EDS spectra for Ti grains in (c). (**f**) Raman data confirms quartz matrix and anatase grains. (**g**) XRD spectra of kaolinite+illite flakes.

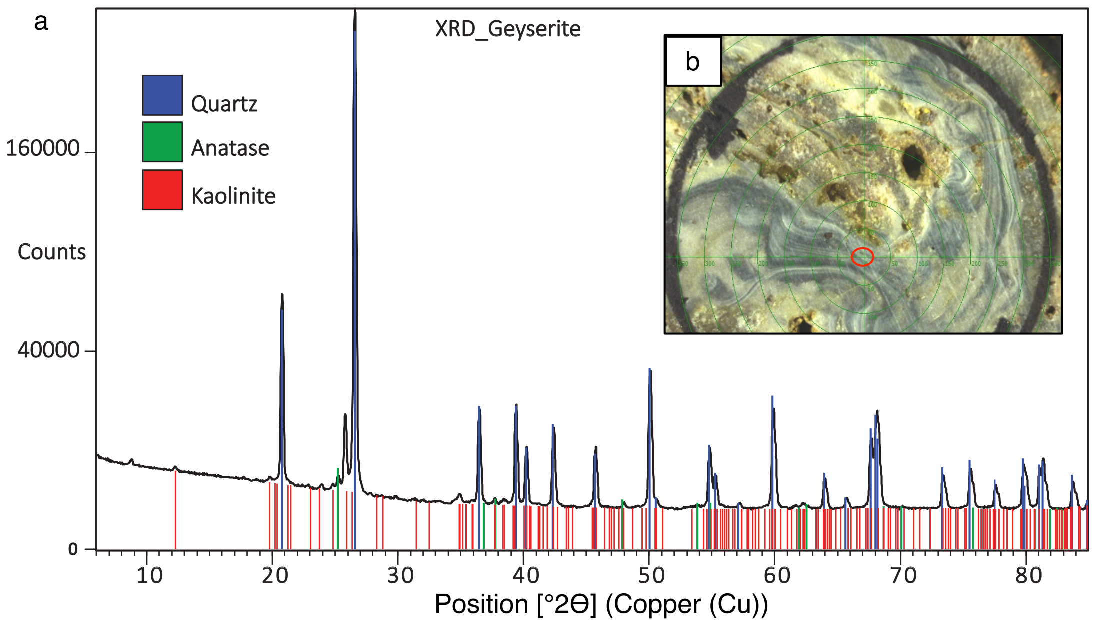

Supplementary Figure 4 | XRD point analysis of Dresser botryoidal geyserite. (a) XRD pattern shows peak data for quartz, anatase, and kaolinite. (**b**) Image of 0.5 mm elliptical analysis area (circled).

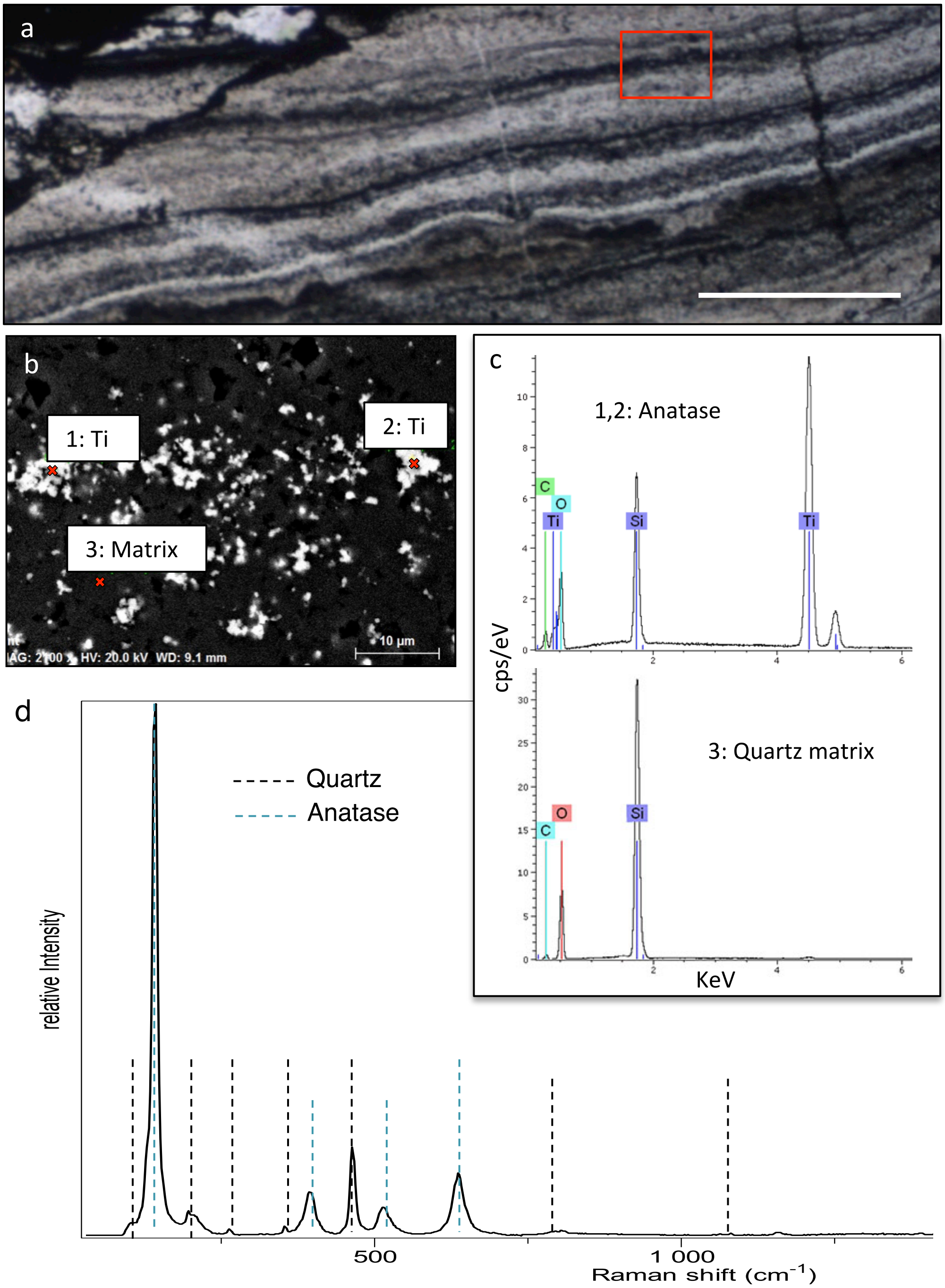

Supplementary Figure 5 | Mineral data of stratiform Dresser geyserite (Location 1S). Scale bar measurements indicated. (**a**) Micrograph of light and dark microlaminations (1 mm), inset box of (**b**) BSE image with point locations for (**c**) EDS spectra showing concentrations of points 1 and 2 with titanium illuminated as bright grains within dark laminae compared with point 3 showing a microquartz matrix. (**d**) Raman spectra confirming TiO_2 (anatase) and SiO_2 (quartz).

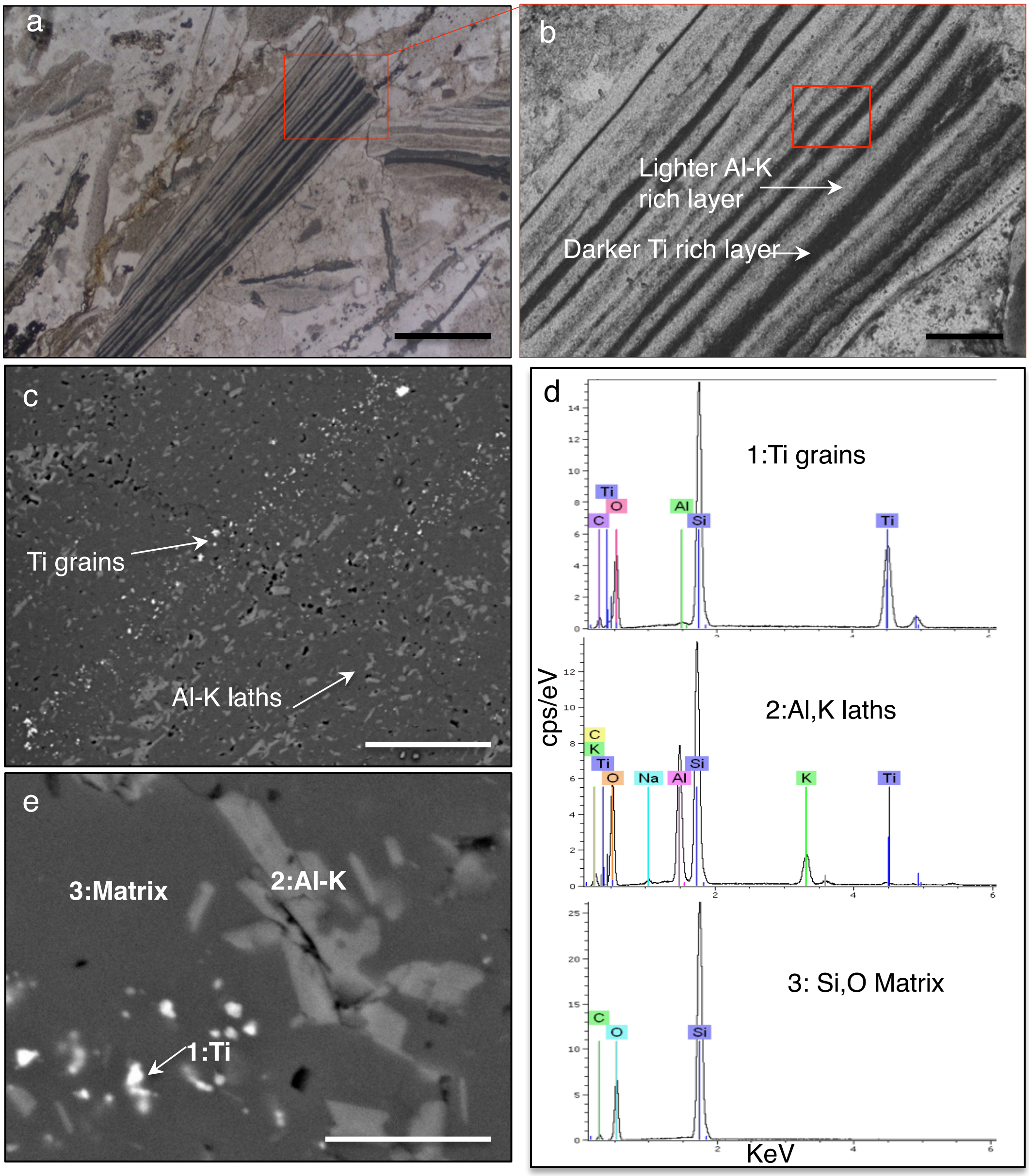

Supplementary Figure 6 | Mineral data of Dresser geyserite 'sintraclasts' (Location 24S). Scale bar measurements indicated. Micrographs (a,b). (**a**) Angular intraclast (1 mm). Inset box of (**b**) showing light (Al-K rich) and dark (Ti-rich) microlaminations. (200 µm). Inset box of (**c**) SEM image showing light grey flakes (Al-K) in light layers and bright (Ti) grains packed in dark layers. (50 µm). (**d**) SEM-EDS spectrographs showing concentration of point locations (**e**) 1 – titanium in bright grains and 2 – Al-K laths, within 3 – a silica rich matrix. (10 µm).

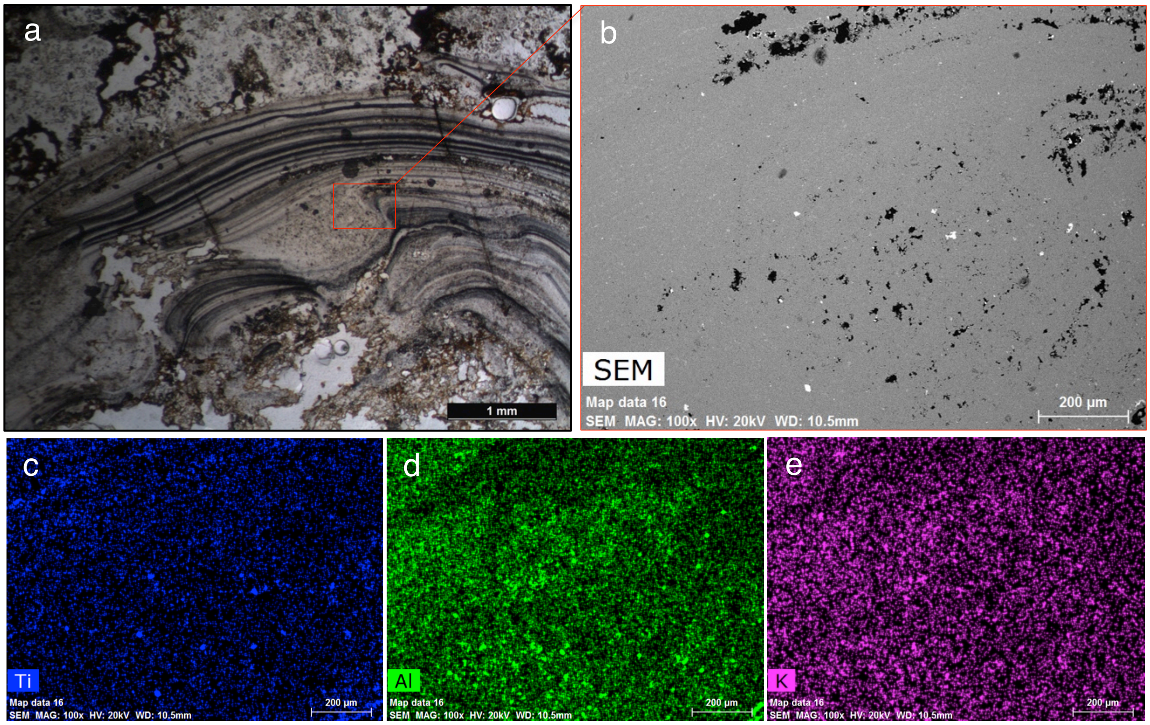

Supplementary Figure 7 | Mineral data of equigranular troughs between Dresser geyserite botryoids. (**a**) Micrograph of equigranular troughs between botryoids. Inset box of (**b**) SEM-EDS image showing portion of equigranular sediment-filled trough. Corresponds to SEM-EDS element map data (**c**) titanium (blue), (**d**) aluminum (green), and (**e**) potassium (magenta).

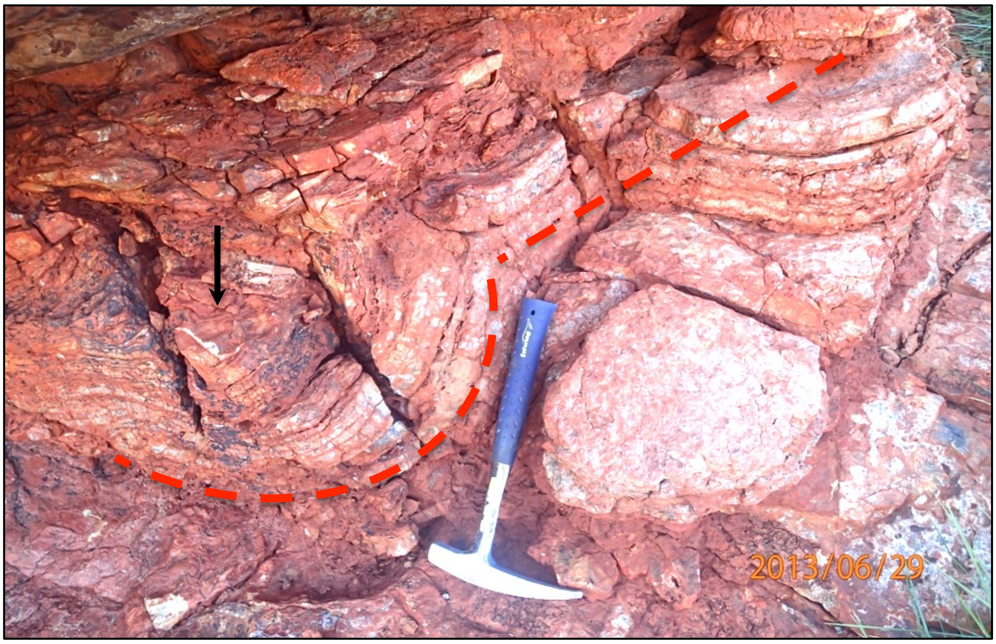

Supplementary Figure 8 | Locality 16N. Outcrop location (arrow) of columnar-botryoidal Dresser geyserite with textural biosignatures. Note the planar to convex barite vein (dashed line) that cuts through the bedding, but terminates beneath the geyserite deposit, representing subsurface hydrothermal fluid flow to the surface.

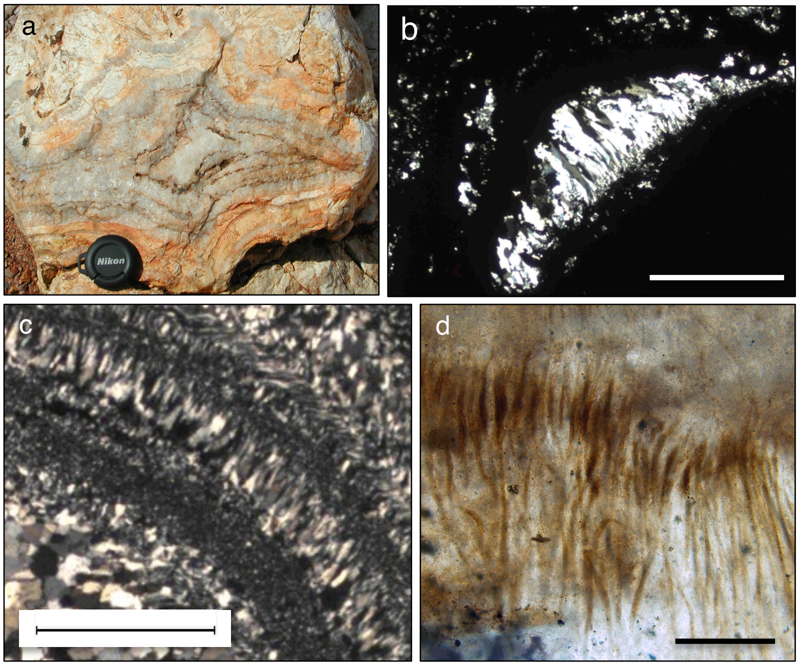

Supplementary Figure 9 | Shearing versus palisade fabrics in the Dresser Formation and modern geothermal settings. Scale bar measurements indicated. Dresser Formation quartz textures; (**a**) Epithermal quartz textures infilling cavity space (Lens cap 2.5 cm). (**b**) Sheared quartz lens beneath domal structure composed of finely layered chert. (1 mm). (**c**) Dresser palisade fabric (Lens cap 2.5 cm) . (**d**) ~150 Ma fossil palisade fabric oriented vertical to bedding, Claudia Sinter, Deseado Massif province, Argentine Patagonia. (80 µm).

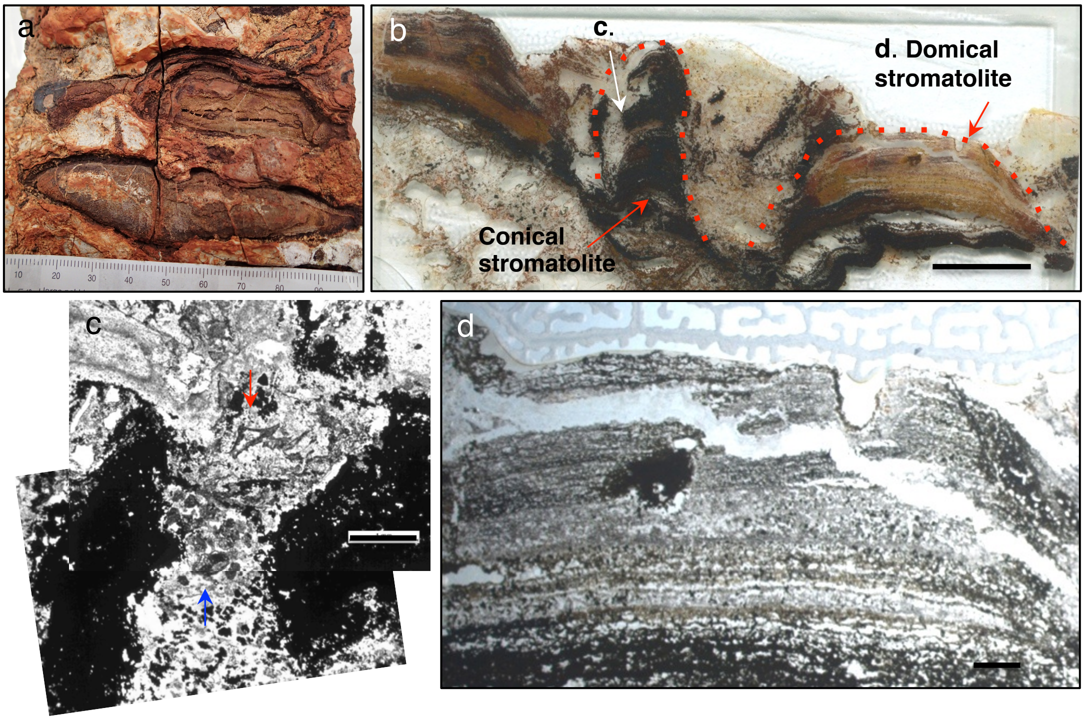

Supplementary Figure 10 | Locality 24S: Elongate domical and conical stromatolites. Scale bar measurements indicated. (**a**) Plan view in outcrop showing irregular, elongate, laminated ovoid stromatolites, 2-3 cm wide x 5-10 cm long. Synoptic relief is < 2.5 cm. They are composed of irregular black, dark brown to creamy brown, opaque laminations. (**b**) Thin section scan of elongate domical and conical stromatolites. (1 mm). Micrographs (c,d). (**c**) Well preserved glass shards (red arrow) and sand sized grains (blue arrow) that infill a crack within the conical stromatolite. (1 mm). (**d**) Microbial laminates with palimpsest fabric within domical stromatolite. (1 mm).
